# Supplementary material for: Effects of Aging, Long-Term and Lifelong Exercise on the Urinary Metabolic Footprint of Rats
Source: Metabolites. 2020 Nov 25;10(12):481. doi: 10.3390/metabo10120481 (PMC7760742; doi:10.3390/metabo10120481)
Supplement: Supplementary file 1 [file metabolites-10-00481-s001.pdf]

Table S1. Metabolites detected in rat urine by LC/MS (in alphabetical order).

No.	Metabolite	Molecular formula	Retention time
1	4-Hydroxyphenyllactate	$C_9H_{10}O_4$	10.15
2	Acetylcarnitine	$C_9H_{17}NO_4$	14.66
3	Adenine	$C_5H_5N_5$	3.90
4	Adenosine	$C_{10}H_{13}N_5O_4$	4.72
5	Alanine	$C_3H_7NO_2$	16.38
6	Arabinose-xylose	$C_5H_{10}O_5$	8.72
7	Betaine	$C_5H_{11}NO_2$	12.56
8	Biotin	$C_{10}H_{16}N_2O_3S$	8.49
9	Cadaverine	$C_5H_{14}N_2$	20.79
10	Choline	$C_5H_{14}NO$	7.25
11	Cotinine	$C_{10}H_{12}N_2O$	1.13
12	Creatine	$C_4H_9N_3O_2$	16.30
13	Creatinine	$C_4H_7N_3O$	4.80
14	Cytidine	$C_9H_{13}N_3O_5$	11.29
15	Cytosine	$C_4H_5N_3O$	7.80
16	Dimethylamine	C_2H_7N	8.20
17	Glucose	$C_6H_{12}O_6$	14.77
18	Glutamine	$C_5H_{10}N_2O_3$	18.12
19	Guanine	$C_5H_5N_5O$	10.40
20	Histamine	$C_5H_9N_3$	13.70
21	Hypotaurine	$C_2H_7NO_2S$	16.02
22	Inosine	$C_{10}H_{12}N_4O_5$	9.48
23	Isoleucine-leucine	$C_6H_{13}NO_2$	13.70
24	Kynurenate	$C_{10}H_7NO_3$	10.12
25	Mannitol	$C_6H_{14}O_6$	14.47
26	Methionine	$C_5H_{11}NO_2S$	14.66
27	Methylamine	CH_5N	10.14
28	Pantothenate	$C_9H_{17}NO_5$	12.82
29	Phenylalanine	$C_9H_{11}NO_2$	12.93
30	Proline	$C_5H_9NO_2$	14.75
31	Putrescine	$C_4H_{12}N_2$	21.00
32	Pyroglutamate	$C_5H_7NO_3$	15.16

33	Pyruvate	$C_3H_4O_3$	7.47
34	Riboflavin	$C_{17}H_{20}N_4O_6$	9.80
35	Sarcosine	$C_3H_7NO_2$	15.30
36	Serine	$C_3H_7NO_3$	18.32
37	Taurine	$C_2H_7NO_3S$	14.73
38	Thiamine	$C_{12}H_{17}N_4OS$	12.02
39	Threonine	$C_4H_9NO_3$	17.10
40	Thymidine	$C_{10}H_{14}N_2O_5$	2.26
41	Trimethylamine-*N*-oxide (TMAO)	C_3H_9NO	13.32
42	Tryptamine	$C_{10}H_{12}N_2$	5.46
43	Tryptophan	$C_{11}H_{12}N_2O_2$	13.02
44	Tyrosine	$C_9H_{11}NO_3$	14.86
45	Uridine	$C_9H_{12}N_2O_6$	4.93
46	Valine	$C_5H_{11}NO_2$	14.40
47	α-Ketoglutarate	$C_5H_6O_5$	16.21
48	γ-Aminobutyrate (GABA)	$C_4H_9NO_2$	17.19

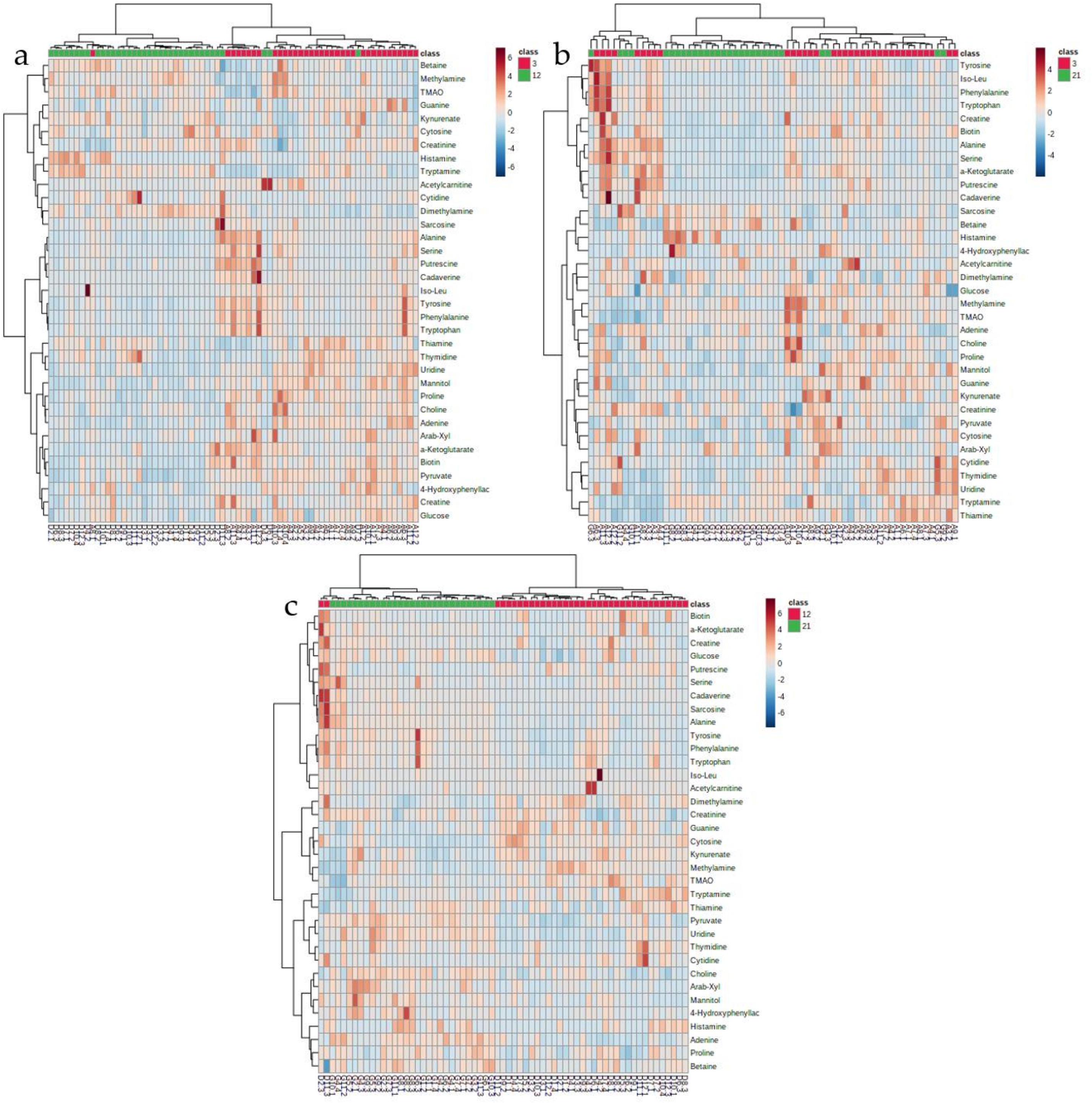

Figure S1. Heat maps of differences in urinary metabolite peak areas between (a) 3 (red heading) and 12 months (green heading), (b) 3 (red heading) and 21 months (green heading), and (c) 12 (red heading) and 21 months (green heading). Rows represent metabolites, and columns represent different urine samples. Shades of red and blue provide a qualitative indication of the magnitude of difference according to the legend. Data were Pareto scaled, and motifs are ranked and sorted by Pearson's hierarchical clustering.

Table S2. Peak areas of urinary metabolites in the samples of group A at three ages. Values are mean ± SD.

Metabolite	3 months (n = 9)	12 months (n = 9)	21 months (n = 7)
4-Hydroxyphenyllactate	0.03 ± 0.01	0.02 ± 0.01	0.05 ± 0.04
Acetylcarnitine	12.74 ± 8.45	6.83 ± 4.72	5.82 ± 2.92
Adenine	0.37 ± 0.05	0.20 ± 0.07	0.35 ± 0.08
Alanine	0.73 ± 0.27	0.39 ± 0.21	0.52 ± 0.10
Arabinose-xylose	0.18 ± 0.07	0.23 ± 0.11	0.21 ± 0.15
Betaine	113.77 ± 19.82	126.88 ± 13.89	122.03 ± 15.06
Biotin	0.19 ± 0.08	0.14 ± 0.16	0.06 ± 0.03
Cadaverine	0.81 ± 0.35	0.21 ± 0.11	0.26 ± 0.03
Choline	0.45 ± 0.33	0.38 ± 0.27	0.51 ± 0.16
Creatine	0.37 ± 0.05	0.20 ± 0.07	0.35 ± 0.08
Creatinine	641.01 ± 43.89	677.01 ± 39.60	673.82 ± 22.27
Cytidine	0.05 ± 0.02	0.07 ± 0.05	0.05 ± 0.02
Cytosine	3.99 ± 0.97	6.30 ± 2.03	2.88 ± 0.70
Dimethylamine	2.05 ± 0.12	2.28 ± 0.29	2.11 ± 0.05
Glucose	0.01 ± 0.00	0.01 ± 0.00	0.01 ± 0.00
Guanine	0.03 ± 0.01	0.03 ± 0.01	0.02 ± 0.00
Histamine	0.24 ± 0.10	0.45 ± 0.14	2.45 ± 3.00
Isoleucine-leucine	113.77 ± 19.82	126.88 ± 13.89	122.03 ± 15.06
Kynurenate	2.46 ± 0.96	4.01 ± 1.68	2.77 ± 1.93
Mannitol	0.03 ± 0.00	0.01 ± 0.01	0.02 ± 0.01
Methylamine	2.51 ± 1.25	2.27 ± 1.26	1.84 ± 0.97
Phenylalanine	1.61 ± 0.21	1.70 ± 0.83	2.04 ± 0.34
Proline	0.03 ± 0.01	0.02 ± 0.01	0.02 ± 0.00
Putrescine	4.96 ± 1.58	5.66 ± 2.02	2.66 ± 0.84
Pyruvate	0.03 ± 0.00	0.01 ± 0.01	0.04 ± 0.01
Sarcosine	0.45 ± 0.33	0.38 ± 0.27	0.51 ± 0.16
Serine	0.38 ± 0.33	0.19 ± 0.13	0.20 ± 0.04
Thymidine	0.18 ± 0.06	0.07 ± 0.02	0.15 ± 0.05
TMAO	64.79 ± 10.75	59.22 ± 8.21	53.99 ± 4.99
Tryptamine	1.16 ± 0.64	1.48 ± 0.73	1.38 ± 0.32
Tryptophan	0.08 ± 0.02	0.07 ± 0.05	0.10 ± 0.03
Tyrosine	0.16 ± 0.07	0.12 ± 0.10	0.19 ± 0.04
Uridine	0.04 ± 0.01	0.02 ± 0.00	0.04 ± 0.01
α-Ketoglutarate	0.19 ± 0.08	0.14 ± 0.16	0.06 ± 0.03

Table S3. Peak areas of urinary metabolites in the samples of group B at three ages. Values are mean ± SD.

Metabolite	3 months (n = 9)	12 months (n = 9)	21 months (n = 9)
4-Hydroxyphenyllactate	0.03 ± 0.01	0.02 ± 0.01	0.04 ± 0.03
Acetylcarnitine	11.58 ± 10.37	7.09 ± 2.49	4.97 ± 2.62
Adenine	0.48 ± 0.16	0.18 ± 0.06	0.34 ± 0.13
Alanine	0.86 ± 0.39	0.19 ± 0.15	0.52 ± 0.12
Arabinose-xylose	0.29 ± 0.12	0.16 ± 0.14	0.40 ± 0.30
Betaine	100.41 ± 13.69	107.52 ± 16.40	118.29 ± 19.58
Biotin	0.22 ± 0.08	0.19 ± 0.14	0.08 ± 0.04
Cadaverine	1.19 ± 0.88	0.12 ± 0.08	0.23 ± 0.04
Choline	0.25 ± 0.10	0.25 ± 0.14	0.51 ± 0.22
Creatine	0.48 ± 0.16	0.18 ± 0.06	0.34 ± 0.13
Creatinine	685.44 ± 34.52	701.34 ± 36.64	676.47 ± 16.43
Cytidine	0.05 ± 0.02	0.05 ± 0.01	0.04 ± 0.02
Cytosine	4.98 ± 0.76	7.04 ± 1.95	3.49 ± 1.68
Dimethylamine	2.04 ± 0.14	2.51 ± 0.21	2.07 ± 0.10
Glucose	0.01 ± 0.00	0.01 ± 0.00	0.01 ± 0.00
Guanine	0.03 ± 0.01	0.03 ± 0.01	0.01 ± 0.00
Histamine	0.16 ± 0.03	0.37 ± 0.30	1.77 ± 1.50
Isoleucine-leucine	100.41 ± 13.69	107.52 ± 16.40	118.29 ± 19.58
Kynurenate	3.08 ± 1.31	4.53 ± 1.19	2.46 ± 1.74
Mannitol	0.03 ± 0.01	0.01 ± 0.01	0.02 ± 0.01
Methylamine	1.50 ± 0.52	2.26 ± 0.43	1.22 ± 0.70
Phenylalanine	2.66 ± 1.37	1.81 ± 0.88	2.43 ± 0.92
Proline	0.03 ± 0.01	0.01 ± 0.00	0.01 ± 0.01
Putrescine	6.59 ± 3.67	3.38 ± 1.57	3.01 ± 1.03
Pyruvate	0.03 ± 0.00	0.02 ± 0.01	0.03 ± 0.01
Sarcosine	0.25 ± 0.10	0.45 ± 0.14	0.51 ± 0.22
Serine	0.43 ± 0.30	0.14 ± 0.08	0.23 ± 0.10
Thymidine	0.17 ± 0.04	0.09 ± 0.05	0.12 ± 0.04
TMAO	49.37 ± 10.50	60.84 ± 6.84	48.83 ± 4.25
Tryptamine	0.98 ± 0.90	2.02 ± 0.87	1.17 ± 0.57
Tryptophan	0.16 ± 0.09	0.06 ± 0.02	0.11 ± 0.03
Tyrosine	0.18 ± 0.07	0.11 ± 0.09	0.20 ± 0.08
Uridine	0.04 ± 0.01	0.02 ± 0.00	0.04 ± 0.01
α-Ketoglutarate	0.22 ± 0.08	0.19 ± 0.14	0.08 ± 0.04

Table S4. Peak areas of urinary metabolites in the samples of group C at three ages. Values are mean ± SD.

Metabolite	3 months (n = 9)	12 months (n = 9)	21 months (n = 8)
4-Hydroxyphenyllactate	0.03 ± 0.02	0.03 ± 0.02	0.06 ± 0.06
Acetylcarnitine	5.97 ± 3.63	7.94 ± 1.62	3.59 ± 1.17
Adenine	0.47 ± 0.13	0.26 ± 0.13	0.39 ± 0.11
Alanine	0.70 ± 0.33	0.37 ± 0.24	0.50 ± 0.07
Arabinose-xylose	0.26 ± 0.12	0.13 ± 0.07	0.50 ± 0.27
Betaine	107.18 ± 24.35	135.28 ± 14.49	127.08 ± 14.33
Biotin	0.30 ± 0.11	0.09 ± 0.06	0.08 ± 0.03
Cadaverine	1.27 ± 1.02	0.17 ± 0.10	0.27 ±0.18
Choline	0.31 ± 0.13	0.19 ± 0.12	0.55 ± 0.15
Creatine	0.47 ± 0.13	0.26 ± 0.13	0.39 ± 0.11
Creatinine	679.18 ± 35.37	667.46 ± 31.30	668.31 ± 24.60
Cytidine	0.09 ± 0.04	0.09 ± 0.07	0.06 ± 0.03
Cytosine	4.91 ± 0.14	5.63 ± 1.43	2.96 ± 0.35
Dimethylamine	2.04 ± 0.23	2.32 ± 0.25	1.95 ± 0.14
Glucose	0.01 ± 0.00	0.01 ± 0.00	0.01 ± 0.00
Guanine	0.03 ± 0.01	0.02 ± 0.01	0.02 ± 0.01
Histamine	7.14 ± 5.76	15.30 ± 11.29	19.68 ± 13.90
Isoleucine-leucine	107.18 ± 24.35	135.28 ± 14.49	127.08 ± 14.33
Kynurenate	4.02 ± 1.39	4.11 ± 0.80	2.91 ± 1.26
Mannitol	0.02 ± 0.01	0.01 ± 0.01	0.02 ± 0.01
Methylamine	1.59 ± 0.45	2.19 ± 0.65	1.42 ± 0.27
Phenylalanine	1.95 ± 0.65	1.86 ± 0.74	1.93 ± 0.24
Proline	0.03 ± 0.01	0.02 ± 0.00	0.01 ± 0.00
Putrescine	6.48 ± 4.95	4.70 ± 1.78	2.22 ± 0.54
Pyruvate	0.03 ± 0.01	0.02 ± 0.00	0.03 ± 0.01
Sarcosine	0.31 ± 0.13	0.19 ± 0.12	0.55 ± 0.15
Serine	0.30 ± 0.22	0.26 ± 0.11	0.17 ± 0.03
Thymidine	0.21 ± 0.09	0.12 ± 0.05	0.16 ± 0.06
TMAO	47.14 ± 9.67	66.00 ± 15.49	51.19 ± 3.58
Tryptamine	1.58 ± 0.93	2.12 ± 0.90	1.51 ± 0.25
Tryptophan	0.11 ± 0.04	0.10 ± 0.07	0.09 ± 0.02
Tyrosine	0.15 ± 0.06	0.12 ± 0.06	0.20 ± 0.03
Uridine	0.04 ± 0.01	0.02 ± 0.01	0.04 ± 0.01
α-Ketoglutarate	0.30 ± 0.11	0.09 ± 0.06	0.08 ± 0.03

Table S5. Peak areas of urinary metabolites in the samples of group D at three ages. Values are mean ± SD.

Metabolite	3 months (n = 9)	12 months (n = 9)	21 months (n = 8)
4-Hydroxyphenyllactate	0.03 ± 0.02	0.02 ± 0.01	0.0 ± 0.03
Acetylcarnitine	7.01 ± 1.67	10.41 ± 3.61	7.15 ± 6.15
Adenine	0.65 ± 0.14	0.22 ± 0.13	0.52 ± 0.12
Alanine	0.16 ± 0.47	0.32 ± 0.09	0.77 ± 0.37
Arabinose-xylose	0.69 ± 0.47	0.07 ± 0.03	0.28 ± 0.22
Betaine	106.05 ± 4.03	120.88 ± 5.83	151.49 ± 15.32
Biotin	0.54 ± 0.22	0.15 ± 0.15	0.07 ± 0.03
Cadaverine	3.20 ± 3.09	0.17 ± 0.15	0.46 ± 0.25
Choline	0.37 ± 0.12	0.42 ± 0.34	0.92 ± 0.35
Creatine	0.65 ± 0.14	0.22 ± 0.13	0.52 ± 0.12
Creatinine	668.32 ± 29.50	690.44 ± 41.64	660.36 ± 31.56
Cytidine	0.08 ± 0.04	0.23 ± 0.15	0.05 ± 0.02
Cytosine	5.55 ± 1.88	5.06 ± 0.16	2.90 ± 0.46
Dimethylamine	1.97 ± 0.16	2.58 ± 0.33	2.01 ± 0.03
Glucose	0.01 ± 0.01	0.01 ± 0.00	0.01 ± 0.00
Guanine	0.02 ± 0.01	0.02 ± 0.01	0.02 ± 0.01
Histamine	4.34 ± 6.30	9.19 ± 12.22	10.53 ± 13.93
Isoleucine-leucine	106.05 ± 4.03	120.88 ± 5.83	151.49 ± 15.32
Kynurenate	2.23 ± 0.52	3.44 ± 1.37	1.58 ± 0.86
Mannitol	0.03 ± 0.01	0.01 ± 0.01	0.02 ± 0.01
Methylamine	2.19 ± 1.81	1.61 ± 0.28	1.05 ± 1.06
Phenylalanine	3.59 ± 1.74	1.54 ± 0.40	2.02 ± 0.75
Proline	0.03 ± 0.01	0.02 ± 0.01	0.02 ± 0.00
Putrescine	10.62 ± 6.99	6.46 ± 4.18	4.17 ± 1.64
Pyruvate	0.04 ± 0.00	0.02 ± 0.01	0.03 ± 0.00
Sarcosine	0.37 ± 0.12	0.42 ± 0.34	0.92 ± 0.35
Serine	0.69 ± 0.41	0.20 ± 0.13	0.37 ± 0.21
Thymidine	0.11 ± 0.01	0.21 ± 0.12	0.17 ± 0.07
TMAO	47.95 ± 30.68	58.65 ± 11.73	44.42 ± 14.62
Tryptamine	0.42 ± 0.35	1.71 ± 0.64	0.80 ± 0.37
Tryptophan	0.22 ± 0.14	0.10 ± 0.07	0.08 ± 0.04
Tyrosine	0.26 ± 0.11	0.09 ± 0.02	0.18 ± 0.06
Uridine	0.04 ± 0.01	0.03 ± 0.01	0.04 ± 0.01
α-Ketoglutarate	0.54 ± 0.22	0.15 ± 0.15	0.07 ± 0.03

Table S6. Summary of univariate analysis, presenting the *p* values that remained significant after FDR correction.

Metabolite	Age	1st half	2nd half	1st x 2nd	Age x 1st	Age x 2nd	Age x 1st x 2nd
Adenine	< 0.001	< 0.001					
Alanine	< 0.001					0.011	
Betaine	< 0.001					0.008	
Biotin	< 0.001				< 0.001		
Choline	< 0.001				0.005		
Creatine	< 0.001					< 0.001	
Creatinine	< 0.001						
Cytidine	0.002	< 0.001			0.008		0.005
Cytosine	< 0.001						
Dimethylamine	< 0.001						
Glucose	< 0.001						
Guanine	< 0.001						0.005
Isoleucine-leucine	< 0.001						
Kynurenate	< 0.001			0.005			
Mannitol	< 0.001						
Methylamine	< 0.001						
Proline	< 0.001						
Pyruvate	< 0.001						
Sarcosine	< 0.001			0.007			
Thymidine	0.002					0.007	
TMAO	< 0.001		0.007				
Tryptamine	< 0.001						
Tryptophan	0.002					0.006	
Tyrosine	< 0.001						
Uridine	< 0.001						
α-Ketoglutarate	< 0.001					0.005	
